# NLRP3 Inflammasome and Inflammatory Diseases

**DOI:** 10.1155/2020/4063562

**Published:** 2020-02-17

**Authors:** Zheng Wang, Simei Zhang, Ying Xiao, Wunai Zhang, Shuai Wu, Tao Qin, Yangyang Yue, Weikun Qian, Li Li

**Affiliations:** ^1^Department of Hepatobiliary Surgery, First Affiliated Hospital, Xi'an Jiaotong University, Xi'an 710061, China; ^2^Department of Ophthalmology, First Affiliated Hospital, Xi'an Jiaotong University, Xi'an 710061, China

## Abstract

Almost all human diseases are strongly associated with inflammation, and a deep understanding of the exact mechanism is helpful for treatment. The NLRP3 inflammasome composed of the NLRP3 protein, procaspase-1, and ASC plays a vital role in regulating inflammation. In this review, NLRP3 regulation and activation, its proinflammatory role in inflammatory diseases, interactions with autophagy, and targeted therapeutic approaches in inflammatory diseases will be summarized.

## 1. Introduction

Inflammasomes, first identified by Martinon and coworkers in 2002 [1–3], are a class of cytosolic complexes of proteins that mediate the activation of potent inflammatory mediators. They are integral parts of the innate immune response against invading pathogens and are activated upon cellular infections or stressors that promote the expression, maturation, and release of a multitude of proinflammatory cytokines, triggering a cascade of inflammatory responses [4, 5]. The nucleotide-binding oligomerization- (NOD-) like receptors (NLRs), a newly identified type of pattern recognition receptors (PRRs), which include Toll-like receptors (TLRs), C-type lectins (CTLs), and galectins, mediate the innate immune response to detect pathogenic microbes and other endogenous or exogenous pathogens [6, 7] and are important components of inflammasomes; they are located within the cytoplasm and recognize pathogen/damage-associated molecular patterns (PAMPs/DAMPs) [8–10]. The NLRs comprise 22 human genes and more mouse genes, and their family members are characterized by the presence of a tripartite structure: a central NOD, which is commonly flanked by C-terminal leucine-rich repeats (LRRs) and a N-terminal caspase recruitment domain (CARD) or pyrin domains (PYDs) [4, 11].

There are 4 known inflammasomes (NLRP1, NLRP3, NLRP4, and Aim2 inflammasomes), and they all contain a PRR that belongs to the NLR family [12, 13]. Among these inflammasomes, the NLRP3 inflammasome plays a pivotal role both in shaping immune responses and regulating the integrity of intestinal homeostasis in many common inflammatory diseases [14, 15]. NLRP3, a multiprotein complex consisting of an NLRP3 scaffold, an adaptor apoptosis speck-like protein (ASC) and the effector procaspase-1, initiates the formation of the inflammasome by interacting with ASC, which recruits and activates procaspase-1 to generate active caspase-1 and then converts the cytokine precursors pro-IL-1*β* and pro-IL-18 into mature and biologically active IL-1*β* and IL-18, respectively. Once activated, the active IL-1*β* and IL-18 will trigger a series of inflammatory responses and pyroptotic cell death [10, 16–18].

The NLRP3 inflammasome is produced by bone marrow-derived macrophages (after stimulation by microbial and nonmicrobial factors such as bacterial toxins, particulate matter, and lipopolysaccharide (LPS)) [8, 19]. The mechanism of NLRP3 activation remains elusive. Several molecular and cellular events have been proposed to describe to be involved in inflammasome activation, including K^+^ efflux, Ca^2+^ signaling, mitochondrial dysfunction, and reactive oxygen species (ROS) production [9]. For example, particulate matter activates the NLRP3 inflammasome by inducing endocytosis and damage to the lysosome membrane, resulting in the release of cathepsin B into the cytosol [20]. Interestingly, the role of ROS and mitochondrial perturbation in NLRP3 inflammasome activation remains controversial and requires further investigation [21–24].

NLRP3 has also been implicated in the pathogenesis of a number of complex diseases, notably including metabolic disorders such as type 2 diabetes [25], atherosclerosis [11, 26–29], obesity, and gout [30]. A role for NLRP3 in diseases of the central nervous system is emerging, including Alzheimer's disease and Parkinson's disease [31, 32]. Abnormal activation of the NLRP3 inflammasome might contribute to intestinal cancer, inflammatory diseases, and autoinflammatory diseases such as keratitis/conjunctivitis [16, 33–36]. In this review, NLRP3 regulation and activation, its proinflammatory role in inflammatory diseases, interactions with autophagy, and targeted therapeutic approaches in inflammatory diseases will be summarized.

## 2. The Role of NLRP3 in Inflammation

Inflammasomes are multiprotein complexes located in macrophages, dendritic cells, and some other immune cells and control the activation of the proteolytic enzyme caspase-1. Caspase-1 then regulates the maturation of IL-1*β* and IL-18 and the subsequent pyroptosis [37]. The NLRP3 inflammasome is composed of the NLRP3 protein, procaspase-1, and ASC [38]. Procaspase-1 is the effector in the NLRP3 inflammasome with a CARD domain. ASC is a bipartite complex containing a PYD and a CARD, which makes it a bridge connecting the sensor NLRP3 and the effector procaspase-1. NLRP3 inflammasome activation is a self-defending mechanism against invading factors and stress. Upon infection and/or injury, inflammasome components assemble and oligomerize, leading to the autocleavage of procaspase-1 to its active form. Activated caspase-1 transforms proinflammatory cytokines into their mature forms, which then participate in the following inflammatory response [39].

The NLRP3 response to stimuli occurs in the trans-Golgi network [40]. The activation of NLRP3 begins with the recognition of the danger or stressor by the sensor PRRs [41]. PAMPs (including microbial nucleic acids, bacterial secretion systems, and components of microbial cell walls) can be sensed by PPRs [42]. In addition, DAMPs (such as ATP and uric acid crystals) can also trigger PPRs [43]. The activation of the NLRP3 inflammasome is a two-stage process. The first stage is the sensing and producing stage, which begins with the recognition of PAMPs and DAMPs by TLRs. In this stage, TLRs recognize various stress factors and activate NF-*κ*B signaling, resulting in elevated production of precursor proteins, including the NLRP3 protein, pro-IL-1*β*, and pro-IL-18 [44]. The second stage is the assembly and effector stage, which begins with the assembly of the NLRP3 inflammasome. The NLRP3 protein, ASC, and procaspase-1 assemble into the mature complex, which then transforms the immature forms of IL-1*β* and IL-18 into their mature forms [45]. IL-1*β* and IL-18 participate in the subsequent inflammatory effect.

NLRP3 is commonly involved in the immune response to bacteria, viruses, fungi, and parasites [42]. In most cases, the recognition of pathogens in the immune response is indirect. TLRs recognize the particular components of the invader and then induce the NLRP3 inflammasome components to be transcribed and assembled. Microbial stimuli, including Bacterial Muramyl Dipeptide (MDP) [46], bacterial RNA [47], and LPS [47], can activate the NLRP3 inflammasome in a TLR-dependent manner, while living microbes, rather than dead microbes, can induce a particular immune response via the Toll/interleukin-1 receptor domain-containing adaptor-inducing interferon-*β*- (TRIF-) dependent recognition by the NLRP3 inflammasome [48].

In addition, various danger signals unrelated to infection can trigger the NLRP3 inflammasome, including ROS, Ca^2+^, nitric oxide (NO), and mitochondrial dysfunction (MtD). The production of ROS in cell has two origins: mitochondria-derived ROS (mtROS) and the cytosolic ROS. The mtROS can act as the second messenger to trigger the activation of inflammasomes after the recognition of PAMPs from microbes or DAMPs [49]. In a research about the muscle wasting, the researchers found that angiotensin II can promote the mtROS production as well as MtD, which further activated NLRP3 inflammasome [50].

The proper function of mitochondria is also crucial for NLRP3 inflammasome activation. Several factors including NO [51] and Ca^2+^ [52] can lead to MtD, which may also trigger the NLRP3 inflammasome activation via the release of oxidized mitochondrial DNA (mtDNA) following the engagement of TLRs [21]. MtD induced by the NLRP3 secondary signal activators can lead to the release of oxidized mtDNA into the cytosol, and then NLRP3 inflammasome is activated by the bondage of mtDNA [53]. Mitophagy, a crucial procedure involved in mitochondrial dynamics, has been reported to has an influence on excessive inflammasome activation. Mitophagy clears damaged mitochondria through a variety of mechanisms, including the activation of the PINK/PARKIN pathway [54], p62 aggregation [55], and SESN2 activation [56].

The endocytosis of silica and asbestos by pulmonary macrophages may activate the NLRP3 inflammasome and ROS signaling, which further leads to silicosis and asbestosis [20]. Similarly, the accumulation of monosodium urate during gout can activate the NLRP3 inflammasome in macrophages [46]. In osteoarthritis, hydroxyapatite crystals are able to activate IL-1*β* and elevate its production through the NLRP3 inflammasome, thus mediating inflammation and joint diseases [57]. In atherosclerosis, the NLRP3 inflammasome drives IL-1*β* release, thus contributing to the progression of atherosclerosis [58]. Similarly, the inhibition of caspase-1 and IL-1*β* activation induced by bone marrow-derived mesenchymal stem cells can suppress the generation of mitochondrial ROS and then inhibit the NLRP3 inflammasome activation [59]. Systemic inflammation has been reported to be related to an overproduction of IL-1*β* and IL-18 [60]. In a mouse model focusing on systemic inflammatory response syndrome, the researchers found that NLRP3 activates the adaptive immune response in mice during acute pancreatitis. This response depends on IL-1*β* and IL-18, but not IL-12 [60]. Similar results have also been observed to support the NLRP3 active effect of IL-18 in an engineered mouse model [61]. However, the exact mechanism by which NLRP3 recognizes DAMPs remains unclear. Studies have reported that K^+^ efflux and Ca^2+^ signaling participate in the activation of the NLRP3 inflammasome [62–66]. Among the reported upstream mechanisms involved in the NLRP3 inflammasome, the generation of mitochondrial ROS is an important one [67]. During ischemia and reperfusion, ethanol, obesity (saturated fatty acids), and ROS can induce NLRP3 inflammasome activation [68–70].

In a research about the HBV infection, researchers found that HBeAg could inhibit the NF-*κ*B pathway and ROS production. This effect prevents LPS from inducing NLRP3 inflammasome activation, without interrupting the intracellular calcium concentration and lysosomal rupture [71]. In addition, in a study of RNA viruses, the production of ROS induced by the RIP1-RIP3 complex activated the NLRP3 inflammasome [72]. NADPH oxidase can produce cytosolic ROS, which is responsible for the activation of the NLRP3 inflammasome [73]; nevertheless, proof to the contrary showed that macrophages lacking NADPH oxidase can exhibit normal activation of the NLRP3 inflammasome [74]. Hence, the importance of ROS in NLRP3 inflammasome function has been widely acknowledged, but the exact mechanism remains to be explored.

## 3. The Crosstalk between NLRP3 and Autophagy

Autophagy is a physiological process that maintains the normal metabolic function and survival of cells. The formation of autophagosome is a feature of autophagy. The first step in autophagosome formation is initiation. The ULK1-Atg13-FIP200 complex is activated and localizes in the endoplasmic reticulum and some other areas. This is followed by a nucleation step driven by class III phosphoinositide 3-kinase complex (consisting of VPS34, VPS15, Beclin 1, ATG14L, and NRBF2) which is activated by ULK1. After the phagophore has almost wrapped the shipment to be degraded, the phagophore stretch and seal the shipment. The elongation step was performed with an Atg5-Atg 12-Atg16L and LC3II-PE conjugate. Then, autophagosome fuses to lysosomes to form autophagolysosomes [75].

Autophagy recycles cellular proteins and damaged organelles to obtain metabolic energy during starvation or stress to modulate cell survival in many diseases. In normoxia, autophagy is essential for maintaining corneal epithelium physiology and cell survival [76]. Additionally, autophagy serves as an essential process in resisting infection by degrading pathogens. In keratitis, the innate immune response, including autophagy, is activated when pathogens adhere to the ocular surface [77]. Interestingly, some viruses (such as HSV1) inhibit autophagy (by binding of the virus protein ICP34.5 to the host protein Beclin 113) and reduce damage [78]. In addition, excessive or abnormal autophagy can lead to cell death. The autophagy of dendritic cells enhanced the activation of CD^4+^ T cells and pathological keratitis, which significantly promoted the occurrence of herpes simplex keratitis [79]. Interfering with autophagy may be able to intervene in this incurable infectious blindness.

Normally, activation of the inflammasome, including NLRP3, triggers an antiviral inflammatory response that clears the virus and cures the inflamed tissue. NLRP3-knockout mice with keratitis induced by HSV1 developed more severe disease than infected wild-type animals, with stromal keratitis lesions occurring earlier and having more angiogenesis; this result may be related to the nuclear translocation of the NLRP3-IRF4 complex in Th2 cells, which promotes the expression of the IL-4, IL-5, and IL-13 genes to fight the HSV1 infection [80, 81]. In addition, the NLRP3/caspase-1/IL-1*β* pathway plays an important role in leukocyte aggregation and fighting infection during Aspergillus fumigatus infection [36]. However, the abnormal activation of the inflammasome will lead to harmful overwhelming inflammation, which may damage the infected tissue. Persistent and abnormal NLRP3 signaling is the basis of many chronic and degenerative diseases, including Stargardt disease type 1 [82], Alzheimer's disease [83], atherosclerosis [84], atrial fibrillation [85], osteoarthritis [86], and cancer [87] (Table 1).

The relationship between autophagy and NLRP3 is complex. Some studies have shown that autophagy could inhibit priming and assembly stages of the NLRP3 inflammasome [88]. In autophagy-deficient cells, including autophagic protein depletion [89], activation of the inflammatory NLRP3 complex is enhanced due to mitochondrial dysfunction such as excessive mitochondrial ROS production and changes in mitochondrial membrane permeability [90], contributed to IL-1*β* and IL-18 secretion. Loss of autophagy/mitophagy can lead to a buildup of cytosolic reactive oxygen species and mitochondrial DNA, which can, in turn, activate immune signaling pathways that ultimately lead to the releases of inflammatory cytokines, including IL-1*α*, IL-1*β*, and IL-18 [91]. In addition, mitophagy can clear damage mitochondria through a variety of mechanisms, including activation of the PINK/PARKIN pathway [91], p62 aggregation [92], and SESN2 activation [91], thereby preventing excessive inflammation activation. Research has shown that resveratrol inhibits NLRP3 activation in macrophages by inhibiting mitochondrial damage and enhancing autophagy [93]. Studies have also shown that autophagosomes can directly encapsulate and degrade inflammasome components, including the linker molecules ASC, NLRP3, and pro-IL-1*β* [94]. However, some studies have also shown that autophagy promotes NLRP3 activation. Zearalenone increases autophagy and triggers NLRP3 resonance activation by promoting NF-*κ*B activation and nuclear translocation, ultimately resulting in cell pyroptosis [95]. In turn, NLRP3 has an effect on autophagy activation. The induction of NLRP3 inflammasomes in macrophages triggers the activation of the G-protein RalB and then the activation of autophagy, which tempers inflammation by eliminating active inflammasomes to prevent a cascade of amplified inflammatory responses [93]. Nevertheless, the inflammation induced by the NLRP3 inflammasome can also inhibit autophagy. In neuritis, the neuroinflammation promoted by NLRP3 inflammatory complexes may be amplified and regulated by a glia maturation factor, thus inhibiting the clearance of the protein aggregates that formed as a result of the autophagic pathway [96]. In nonalcoholic steatohepatitis, NLRP3 and caspase-1 can inhibit autophagy by regulating the PINK/PARKIN pathway [91]. Additionally, the NLRP3 inflammasome inhibitor MCC950 can activate autophagy and PPAR*α* through mTOR inhibition [97]. In conclusion, the complex relationship between NLRP3 and autophagy needs more research to provide new ideas for clinical treatment.

## 4. The Therapeutic Prospect of NLRP3 on Related Diseases

In clinical settings, the NLRP3 inflammasome is upregulated in myocardial fibroblasts mainly during acute myocardial infarction (AMI) [98]. van Hout et al. [99] also proved that the inflammasome can be inhibited by MCC950 in large animal AMI models. In addition, the immune complexes in systemic lupus erythematosus (SLE) patients can trigger the NLRP3 inflammasome, activate macrophages, and cause cell and tissue damage [100]. A recent study [101] has shown that citral can inhibit the expression of pro-IL-1*β* mediated by endotoxin and the activation of the NLRP3 inflammasome mediated by ATP, which is intriguing for the treatment of SLE. Moreover, activation of the NLRP3 inflammasome also plays an important role in the nonspecific inflammation of inflammatory bowel disease (IBD). It is noteworthy that Villani et al. [102] found that the SNP rs10733113 in the NLRP3 gene region is a Crohn's disease susceptibility gene. Subsequently, Lewis et al. [103] also reported that men carrying the c10x motif in card8, Q705k in NLRP3, and wild-type NOD2 showed susceptibility to Crohn's disease. In addition, a recent study [104] has shown that dysfunctional CARD8 mutations can also activate the NLRP3 inflammasome and contribute to the occurrence of Crohn's disease. Clarification of the exact physiological mechanism of the NLRP3 inflammasome will undoubtedly guide the development of effective treatments for IBD in the future.

NLRP3 inflammasomes are of great importance to therapies targeting inflammation due to their critical role in regulating inflammation. In many bacterial infections, pathogens activate NLRP3-based inflammation through the secretion of pore-forming toxins by Staphylococcus aureus [105]. Vibrio cholerae secretes toxins to activate NLRP3 similar to Staphylococcus aureus. In vivo, mice lacking inflammatory components showed that caspase-1 and ASC had protective effects against Vibrio cholerae infection [106]. NLRP3 was beneficial for mice during pneumonia caused by Streptococcus pneumoniae, and NLRP3^−/−^ mice had higher bacterial load and higher mortality than wild-type mice [107]. The NLRP3 inflammasome can also be activated by viruses, such as influenza A, through the recognition of viral RNA [108]. Recent studies [109, 110] have shown that the NLRP3 inflammasome can be activated by superficial fungi such as T. schoenleinii and M. canis or their components through direct or indirect pathways to produce active inflammatory factors, which play an important role in host immunity. Currently, it has been found that the mechanisms against infection of nonsuperficial fungi may be related to the NLRP3 inflammasome [111–113]. NLRP3 can recognize Candida albicans, activate the NLRP3 inflammation complex, and induce pro-IL-1*β* processing, maturation, and secretion [114, 115]. The mortality rate of NLRP3 or ASC gene-deficient mice after infection with Cryptococcus neoformans was higher than that of wild-type mice, and the bacterial load in the lung tissues of NLRP3-deficient mice was significantly higher than that of wild-type mice [116]. These results showed that the NLRP3 inflammasome plays an important role in the host response to cryptococcal infection.

In eye diseases, the NLRP3 inflammasome has been shown to contribute to diabetic retinopathy [117], acute glaucoma [118], age-related macular degeneration [119], Behcet's syndrome, and dry eye disease [120]. In addition, in a mouse model of Pseudomonas aeruginosa keratitis, the inhibition of caspase-1 and the killing of bacteria by ciprofloxacin reduced the severity of corneal inflammation [121]. Another study showed that in a mouse model of keratitis, the level of IL-1 increased starting at 4 h after infection [122]. Treatment with the IL-1 receptor antagonist anakinra has also proven successful in the treatment of scleritis and episcleritis in the context of different rheumatic conditions [123].

To treat NLRP3-related diseases, researchers have found several inhibitors of NLRP3 or IL-1*β*, including direct inhibitors of NLRP3 proteins such as MCC950 [124, 125], 3,4-methylenedioxy-*β*-nitrostyrene (MNS) [126], CY-09 [127], and OLT1177 [128], indirect inhibitors such as glyburide [129], 16673-34-0, and JC124 [130], and inhibitors of components of the complex such as *β*-hydroxybutyrate (BHB) [70], parthenolide, and bay 11-7082 [131] (Table 2). The NLRP3 inflammasome can also produce IL-18, which leads to physical disorders [132]. Compared to blocking IL-1*β*, specific targeting with NLRP3 inhibitors may be a good choice for related diseases [133]. However, the Food and Drug Administration (FDA) does not currently approve these drugs. Future research should focus on the development of structure-oriented direct inhibitors to improve the specificity and effectiveness.

## 5. Discussion

NLRP3 plays a vital role in various inflammatory diseases by altering immune responses or regulating the integrity of intestinal homeostasis. ROS, K^+^ efflux, and Ca^2+^ signaling have been suggested to activate NLRP3 [9], but the specific mechanism remains unclear. Particularly, the role of ROS in NLRP3 inflammasome activation remains controversial, and it has been revealed that the cytosolic ROS induced by NADPH is responsible for the activation of the NLRP3 inflammasome [73]. However, other studies have shown that macrophages lacking NADPH oxidase exhibit normal activation of the NLRP3 inflammasome [74]. Therefore, we can conclude that the function of ROS is undetermined in the NLRP3 inflammasome, and more precise research about the mechanism is necessary.

**Figure 1:**
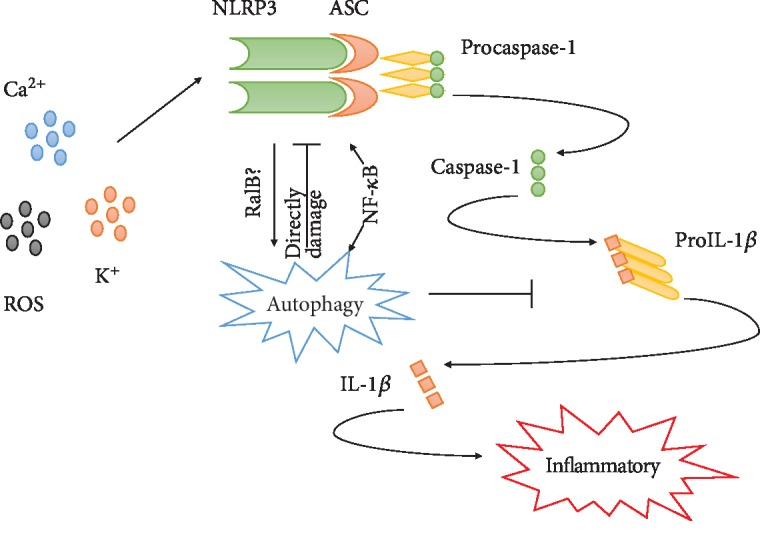
NLRP3 inflammasome-mediated inflammation and autophagy have complex and bidirectional regulatory effects. After being stimulated by Ca^2+^, K^+^, or ROS, the NLRP3 inflammasome is activated and recruits and activates procaspase-1 to generate active caspase-1, which then converts the cytokine precursor pro-IL-1*β* or other proinflammatory cytokines into mature and biologically active forms and triggers a series of inflammatory responses and pyroptotic cell death. However, this process can be regulated and interrupted by autophagy via damage of NLRP3 inflammasome; however, NLRP3 can promote cell autophagy via activation of the G-protein RalB. Interestingly, the relationship between NLRP3 and autophagy is not definitively understood, and there have also been reports that contradict the above statement such as NF-*κ*B activation can modulate the NLRP3 and autophagy in same direction.

The NLRP3 inflammasome is composed of the NLRP3 protein, procaspase-1, and ASC [134] and can generate active caspase-1 and then convert the cytokine precursors pro-IL-1*β* and pro-IL-18 into mature and biologically active IL-1*β* and IL-18, respectively. Ultimately, active IL-1*β* and IL-18 trigger a series of inflammatory responses and pyroptotic cell death [17, 18, 135]. As an important physiological process, autophagy is also strongly associated with the NLRP3 inflammasome. Many studies have shown that autophagosomes can directly encapsulate and degrade inflammasome components, including the linker molecules ASC, NLRP3, and pro-IL-1*β* [90, 93, 94]. Nevertheless, other researchers have demonstrated that autophagy can promote the activation of the NLRP3 inflammasome and that NLRP3 also triggers autophagy by activating the G-protein RalB in turn [95–97]. NLRP3 and autophagy have a complex relationship, and an exploration of this relationship will be helpful for understanding the mechanism of inflammation (Figure 1).

The NLRP3 inflammasome is considered a promising target for the treatment of many diseases associated with inflammation. In AMI, SLE, IBD, Crohn's disease, bacterial infections, eye diseases, etc., the NLRP3 inflammasome plays a critical role in regulating pathological processes [98, 100, 102–105, 119, 136]. Although several inhibitors of the NLRP3 inflammasome have been developed, they have not been approved by the FDA and more basic and clinical research to confirm the curative effects is necessary. With in-depth research on the mechanism of the NLRP3 inflammasome, we believe that a more exact mechanism of the NLRP3 inflammasome itself and its relationship with autophagy will be uncovered and that more specific and effective inhibitors will be exploited.

## Figures and Tables

**Table 1:** Role of NLRP3 inflammasome in disease.

Disease	Responsible factor	Effect	Ref
Aspergillus fumigatus keratitis	NLRP3, caspase-1, and IL-1*β*	Pannexin 1 channels play important roles in the regulation of progression and leucocyte aggregation during corneal *A. fumigatus* infection via the NLRP3/caspase-1/IL-1*β* pathway.	[36]
Stargardt disease type 1	NLRP3, ROS, IL-1*β*, and IL-18	Aberrant buildup of atRAL promotes the death of RPE cells via NLRP3 inflammasome activation.	[82]
Alzheimer's disease	NLRP3, caspase-1, and IL-1*β*	Strongly enhanced the active NLRP3/caspase-1 axis in human mild cognitive impairment and brains with Alzheimer's disease.	[83]
Atherosclerosis	NLRP3	NLRP3 was overexpressed in aorta of patients with coronary atherosclerosis.	[84]
Atrial fibrillation	NLRP3	The inhibition of NLRP3 as a potential novel AF therapy approach.	[85]
Osteoarthritis	NLRP3, caspase-1, and IL-1*β*	Inhibition to the release of inflammasome NLRP3 exerts protection on osteoarthritis leading to the downregulation of inflammatory cytokines.	[85]
Cancer	NLRP3, caspase-1, IL-1*β*, and IL-18	Dysregulation of NLRP3 inflammasome activation is involved in tumor pathogenesis.	[87]

**Table 2:** Inhibitors of NLRP3 pathways as well as their effects in cell cultures, animal models, or patients of inflammatory diseases.

Inhibitors	Molecular mechanism	Cell/animal model/patients	Ref
MCC950	Block the ATPase domain of NLRP3 and inhibit the activation of typical and atypical NLRP3 inflammasome	Autoimmune encephalomyelitis Cryopyrin-associated periodic syndrome Muckle-Wells syndrome	[124]

MNS	Bind to the LRR and NACHT domains and suppress ATPase activity of NLRP3	Bone marrow-derived macrophages	[91]

CY-09	Inhibit NLRP3 ATPase activity	Cryopyrin-associated autoinflammatory syndrome Type 2 diabetes Synovial fluid cells from gout patients	[137]

OLT1177	Inhibit NLRP3 ATPase activity and block canonical and noncanonical activation of NLRP3 inflammasome	Human blood-derived macrophages Human blood neutrophils Monocytes isolated from patients with cryopyrin-associated periodic syndrome Spleen cells from mice	[128]

Glyburide	Inhibit ATP-sensitive K^+^ channels, act as downstream of the P2X7 receptor, and inhibit ASC aggregation	Bone marrow-derived macrophages Familial cold-associated autoinflammatory syndrome patients	[129]

16673-34-0	Interfere the downstream of NLRP3 conformational changes and bind to ASC	Acute myocardial infarction	[138]

JC124	Block ASC aggregation, caspase-1 activation, and IL-1*β* secretion	Acute myocardial infarction Alzheimer's disease	[139] [140]

BHB	Inhibit K^+^ efflux and block ASC aggregation	Muckle-Wells syndrome Familial cold autoinflammatory syndrome Urate crystal-induced peritonitis	[70]

Parthenolide	Inhibit caspase-1 activation and NLRP3 ATPase activity	Bone marrow-derived macrophages Cystic fibrosis	[131] [141]

Bay 11-7082	Alkylation of cysteine residues of the NLRP3 ATPase region	Psoriasis-like dermatitis Diabetic nephropathy	[142] [143]
